# Muhammad Shahrur's millenial interpretation of women’s issues

**DOI:** 10.12688/f1000research.125653.1

**Published:** 2023-01-10

**Authors:** Saifuddin Herlambang

**Affiliations:** 1 Fakultas Ushuluddin, Adab dan Dakwah, State Institute for Islamic Studies (IAIN) Pontianak, Pontianak, West Borneo, 78243, Indonesia

**Keywords:** Women, Millennial Commentary, Muhammad Shahrur, Theory of limits

## Abstract

Background: This study examines Muhammad Shahrur's theory of limits and women’s issues. This theory adjusts the muhkamat verses to remain relevant to sociocultural conditions while remining within the jurisdiction of Allah SWT. Shahrur's approach conveyed women as different from traditional ulema, and controversial. Therefore, it is necessary to describe Shahrur's theory and interpretation on women issues.

Methods: This was an exploratory bibliographic study using descriptive-hermeneutic analysis. Two of Shahrur's books were selected and five chapters from *Al-Kitāb wa Al-Qur’ān, Qirā’ah Mu’āshirah* and five chapters from *Nahw Ushūl al-Jadīdah Li al-Fiqh al-Islāmy; Fiqh al-Mar'ah* .

Results: Shahrur’s interpretations on women issues are: a) relationships between teenagers of the opposite sex without marriage or living together is “ *hala*l” if it follows their will, without a contract, being accompanied by syecih or permission, b) The maximum limit of hijab is to cover the entire body except the face and palms. In contrast, the minimum limit covers the juyūb, including cleavage under the armpits, body parts, genitals, and buttocks. Apart from these, it does not include intimate parts and adapts to the community. c) women's intimate parts are shown only to the seven groups; brothers, fathers, children of a sibling, parents of one's wives and their children, d) Polygamy has both an upper limit and lower limit. Shahrur allows polygamy under two conditions: widows with children whose husbands left them to protect them, polygamy has two limitations, limit for quantity and limit for quality, e) the law of adultery has a lower and upper limit.

Conclusion: According to Shahrur, women's issues are divided into four limits, sometimes they are at the upper limit and sometimes at the lower limit. Shahrur's linguistic approach finally led him to draw a conclusion that the product of Islamic law is highly dependent on the sociocultural context.

## Introduction

In the public domain, the condition of women and men -- when compared to the periods of colonialism and imperialism -- is now much better. Many women also get into politics, have a career, are highly educated, and so on. But this does not mean that it guarantees justice for women, especially at the religious discursive level. Today, in Indonesia as a country where the religion embraced by the majority of the population is Islam, unfair treatment of women by men, in the form of discrimination and violence, still occurs. It is true that the state must be present. However, it is too difficult for the state to deal with it alone. It needs religious support and some kind of religious commentary and or *fiqh* in favor of women.

Devi Asmarani, a women's activist, in a Focus Group Discussion (FGD) on Women and Gender Equality, in Jakarta, on 8 October 2018, said that currently there are at least 400 Regional Regulations ( *Perda*) that discriminate against women. “This discrimination is, for example, in the form of a Regional Regulation that limits the way women dress and limiting the time for women's activities outside the home.” According to her, the clash of views between religion and politics is one of the factors forming discriminatory attitudes towards women. Opportunities for women in Indonesia, to become regional heads or members of the Legislative, are very small, especially in areas that apply Islamic law, where discrimination still occurs. ^1^

In addition to discrimination, the cases of violence against women in Indonesia are in fact still high in number. This is revealed from data released by the National Commission for Eradication of Violence against Women (Komnas perempuan) in 2015. In the data, it was recorded that there were at least 16,217 cases that went to Komnas Perempuan. A total of 11,207 cases of which occurred in households. A total of 6,725 out of 11,207 cases of violence against women were experienced by wives. Meanwhile, the other 2,734 cases were experienced by women whose status was girlfriend. There were 930 cases of violence against young women. The data on violence against women released by Komnas Perempuan is far less than the data presented by the Religious Courts-Religious Judiciary Agency (PA-BADILAG). According to data from PA-BADILAG, violence against women reached 321,752 cases. Thus, when combined with data from Komnas Perempuan, violence against women reached 321,752 cases. ^2^

The leader of the Cirebon Arjawinangun Islamic Boarding School, KH Husein Muhammad, argued that the rise of cases of violence against women is partly due to the wrong interpretation of Islamic teaching text and that is a reason for people to commit violence in the name of religion. ^3^ According to Husein, one of the texts that is often misinterpreted so that it is considered discriminatory is Surah An Nisa verse 34. The Surah is often interpreted as follows:

*Men are the caretakers of women, as men have been provisioned by Allah over women and tasked with supporting them financially. And righteous women are devoutly obedient and, when alone, protective of what Allah has entrusted them with. And if you sense ill-conduct from your women, advise them ˹first˺, ˹if they persist,˺ do not share their beds, ˹but if they still persist,˺ then cahtise them. But if they change their ways, do not be unjust to them. Surely Allah is Most High, All-Great.* ^4^

In the verse, according to Husein, there is the word “chastise” which is used as the basis for husbands to commit violence against their wives. In fact, Husein continued, the interpretation is not like that, it is not only women who are blamed, but men who are at fault must also be punished according to Husein. ^5^ Such an interpretation also emphasizes the position of women as inferiors who must always obey men. Otherwise, men feel entitled to violence. Husein emphasized that Islam is not a discriminatory religion, but a wrong understanding of Islamic texts often gives rise to discriminatory views. This situation occurs partly because the interpretation of Islamic texts is not made by women due to social construction that places women's roles and functions only in the domestic area. ^6^

If in today's millennial era, Islam gets more attention in religious studies. I think this is not only due to the development and global impact of the world's Muslim population (as mentioned by Richard C. Martin), ^7^ or because of the emergence of religious awareness in the public domain in the modern era (as described by Jose Casanova), ^8^ but in fact because the teachings of Islam adhered to by Muslims themselves are in fact very problematic; not yet in synergy with the needs of the times. Oftentimes, Muslims even produce views that are opposite to the normative views of Islam which highly respect human rights. Therefore, in this context, many Muslim reformers, who then called for the importance of reforming religious (Islamic) thoughts, say for example: Mohammaed Abed Aljabiri, ^9^ Khaled M. Abou el-Fadl, ^10^ Abdul Karim Soroush, ^11^ Abdullah Saeed, ^12^ and also Muhammad Shahrur. ^13^

Especially the last one, Muhammad Shahrur is a Muslim thinker who was chosen as the focus of study in this paper. It is important to discuss Shahrur's thoughts as other contemporary Muslim thinkers, in order to reform Islamic understandings which in this century are less relevant and do not synergize with universal human rights trends. The situation of women in Muslim countries or where the majority of the population is Muslim (such as in Indonesia) who live their daily lives in a circle of discriminatory religious policies, became the focus of Muslim reformers including Shahrur, who in turn sparked many important projects for a number of Islamic *harakah* (Muslim movement). All *harakahs* agree that reform is important, especially as times continue to change, as well as the need to produce actual religious thoughts, but in the applicable domain there are sometimes differences of opinion between one another. It is reasonable because the methodologies or approaches used are not the same or vary. ^14^ But at least, in general, there are two types of *harakah Islamiyyah* (Muslim movement) that emerge in this context: first, “leftists” who develop their thought based on liberal Western methodology; and “rightists” in the sense of guarding reform based on the thoughts of classical Muslim scholars. ^15^

Muhammad Shahrur, in this context as a thinker who provides many ideas about the reform of Islamic thought, especially in the field of commentary, tends to be leftist and liberal. His ideas of women in the study of commentary, for example, have sparked a lot of debates, especially when viewed from the perspective of classical *fiqh* (Islamic Jurisprudence). Based on the background above, this study focused on describing Muhammad Shahrur's limits theory, which defines a lower limit ( *al-hadd al-adna*/minimum) and an upper limit ( *al-hadd al-a'la*/maximum) and the interpretation of this limit theory on the relationship between teenagers, hijab, women *aurat* (vital intimate), and polygamy on women issues in the millennial era.

## Methods

### Study approach

This paper was deliberately written to discuss Muhammad Shahrur's commentary on women. This study used a literature review approach, which analyzed the sources in a descriptive-hermeneutic manner. Tafseer/books from millenial interpreters discussing women were considered eligible for the study. I used Pontianak State Institute for Islamic Studies library and the internet to conduct the search using the library information system with ‘women interpretation’ as keywords.

### Search strategy

I found several books of tafseer but few discuss women in the millenial era. These were: *al-Kitâb wa al-Qur’ân: Qirâ’ah Mu’âshirah, al-Kitâb wa al-Qur’ân: Qirâ’ah Mu’âshirah (1990), Dirasat al-Islamiyat al-Mu'ashirah fi al-Dawlah wa al-Mujtama' (1996), al-Islâm wa al-Imân: Manzhûmah al-Qiyam (1996),* and *Nahwa Ushûl Jadîdah Li al-Fiqh al-Islâmy: Fiqh al-Mar’ah (2000).* I then searched books eligible for millenial interpretation about women’s issues. Two Shahrur books met all the criteria above. The main sources of this study are two books written by Muhammad Shahrur, namely: *Al-Kitāb wa Al-Qur’ān*, *Qirā’ah Mu’āshirah*, and *Nahw Ushūl al-Jadīdah Li al-Fiqh al-Islāmy; Fiqh al-Mar’ah.* I then chose chapters that discuss women in both books. The search strategy involved looking at the table of contents in both books for women’s issues. Furthermore, a list of the most suitable items according to the aims of this study was obtained. The list of materials/items is as follows;

### Data collection

The data were collected through the following steps; 1) bookmarking and recording narrative texts that serve as research data, 2) Making an inventory of words or texts related to women's issues. Shahrur's book entitled *Al-Kitāb wa Al-Qur’ān, Qirā’ah Mu’āshirah* consists of 4 chapters with 729 pages. Chapter 1 consists of 5 sections, chapter 2 consists of 4 sections, chapter 3 consists of 3 sections, and chapter 4 consists of 2 sections with an addition of a closing chapter consisting of 5 sections. The book contains women's issues related to limits (page 453), polygamy (page 597), clothing (page 604), male and female family relationships (page 604). Meanwhile, Shahrur's book entitled *Nahw Ushūl al-Jadīdah Li al-Fiqh al-Islāmy; Fiqh al-Mar’ah* consists of 6 sections with 381 pages; in these sections there are texts related to women's issues, namely; polygamy (page 301), marriage (page 307), clothing (page 331-343), jewelry (362-365), and dress code for females (page 374) (see Tables 1 and 2).

**Table 1.** Women’s issues on *Al-Kitāb wa Al-Qur’ān*, *Qirā’ah Mu’āshirah.*

No	chapter	chapter	pages
1.	**Limits of legislation and worship**	الحدود فى التشريع والعبادات	453-467
2.	**Polygamy**	التعدد الزوجات	597
3.	**Men and woman clothes**	لباس الرجل والمرأة	604
4.	**Familiy relationship between man and woman**	العلاقة العائلية بين الرجل والمرأة	604
5.	**The relationship between men and women**	العلاقة بين الرجل والمرأة	628

**Table 2.** Women’s issues on *Nahw Ushūl al-Jadīdah Li al-Fiqh al-Islāmy; Fiqh al-Mar’ah.*

No	chapter	chapter	pages
1.	Polygamy	التعددية الزيجية	301
2.	Marital	الزوجية	307
3.	Clothes	لباس	331-343
4.	Jewerly	زينة المرأة	362-365
5.	Rules of women clothes	حكم لباس النساء	374

### Analysis

Hermeneutics is a process of elaborating in terms of content and meaning what is hidden behind the text. ^16^ This study aimed to interpret women’s issues in two of Shahrur's books, using hermeneutics data analysis. Paler states that hermeneutics is a process of elaborating in terms of content and meaning what is hidden behind the text. ^17^ Michelle Byrne also states that the hermeneutics method is used to understand and interpret the text meaning, ^18^ Based on Gadamer's elaborate understanding, interpretation and application, the analyses are inherent in the interpretation. ^19^ After examining passages on polygamy, marriage, clothing, jewelry and dress code for females in Shahrur's books, I interpreted and analyzed these texts, and finally compared them to the verses of the Holy Qur'an and the application of Shahrur's theory of limits.

## Results and discussion

### Muhammad Shahrur as a Islamic millennial thinker and interpreter

Muhammad Shahrur is known as a millennial thinker, whom A. Lutfi Syaukanie classified as leftist and liberal. ^20^ He was born in the Salihiyyah intersection, Damascus, Syria, on 11 April 1938. At the age of 19, Shahrur obtained a *Tsanawiyah* diploma from the Abdurrahman al-Kawakibi madrasa in 1957. ^21^ However, these schools are not religious schools. In other words, Shahrur did not receive sufficient religious education in his childhood and youth; at least in a formal sense. Syahrûr earned his Master's degree and three years later, in 1972, he successfully completed his Doctoral program. In the same year he was officially appointed as a lecturer in the Faculty of Civil Engineering at the University of Damascus and teaches courses in Land Mechanics and Geology *(Mikanika at-Turbât wa al-Mansya’ât al-Ardhiyyah).* ^22^

During his career as a lecturer, Shahrur wrote several books which were not really his specialty. He made his best effort to combine his knowledge with Islamic teachings, especially with the Qur’an. After finding ideas, he began to combine them with verses from the Qur’an. His thoughts related to Islamic law, where he argues that there is an upper limit and a lower limit in Islamic law, finally became known as the theory of limits. While teaching, Shahrur actively wrote books. He tried to interpret the verses of the Qur’an, and in addition, he also proposed the theory of limits in law which basically states that law has an upper and lower limit. In his *Al-Kitab*, Shahrur derives the word Al-Quran from Al-Kitab, where the origin of the word is *ka-ta-ba.* It means an effort made by someone who collects various themes and composes sentences and links one event to another to form a complete piece of writing. ^23^ Therefore, the revelation received by Prophet Muhammad (p.b.u.h) is called a *kitab* [book] because it contains various themes. In this book, Shahrur was heavily involved in linguistic interpretation. He divided the book into two sections: a) the first section is the *kitab* related to human behavior such as prayer, alms, fasting, pilgrimage. Human capacity to carry out this first section of the book is limited, and therefore humans have the ability to choose or be detached from it; b) the second section is the *kitab* in the form of *ma'rifah* which consists of natural law and human life such as death, the end of the world, etc.

In this context, it is necessary to understand that Shahrur did not join any Islamic institution, nor did he take any formal training or obtain a certificate in Islamic sciences. The works he has written, among others, are *Al-Kitāb wa Al-Qur'ān–Qirā'ah Mu'āshirah (1990), Al-Daulah wa al-Mujtama'(1994), Al-Islām wa al-Īmān–Manzhūmah al- Qiyam-(1996), Nahw Ushl al-Jadīdah Li al-Fiqh al-Islāmy (2000), and Tajfīf Manābi' al-Irhāb (2008).* Of these works, the ones that have received the most attention are *Al-Kitāb wa Al-Qur'ān –Qirā'ah Mu'āshirah* (Contemporary Study of the Book and Al-Quran) and *Nahw Ushl al-Jadīdah Li al-Fiqh al-Islāmy* (Contemporary Islamic Fiqh Methodology). His monumental work, *Al Kitab wa Al Qur'an, Qira'ah Mu'ashirah* (Contemporary Study of the Book and Al-Quran) is his greatest work. ^24^

He often involved himself in issues of shari'a liberalization and deconstruction of the the Qur'anic commentary. Several Islamic laws and the rules of the science of commentary and *ushul fiqh* were deconstructed with technical knowledge and relying on their Arabic origins. His environmental background, both education and socialization, also influenced his way of thinking.

### Muhammad Shahrur’s millennial commentary approach

The term millennial commentary, of course, is not a term coined by Shahrur, but a term that the author attaches to Shahrur's thoughts on commentary. In my humble opinion, this term is appropriate after reading Shahrur's views on commentary, which are relevant to be applied with the millennial spirit of this era, an era where all forms of culture and lifestyle meet and influence each other. In this context, Shahrur himself agreed that legal products must be able to synergize with the socio-cultural context, including the times.

Shahrur came to the realization that the outcome of Islamic law is greatly influenced by the socio-cultural setting as a result of his language approach to the study of commentary. In order to bring the texts of the Qur'an—the foundation of fiqh—into line with the realities of society everywhere and at any time, Shahrur thought it necessary to reinterpret them. Shahrur mistrusted the Sunnah al-Nabawiyyah as a source of law due to his emphasis on the Qur'anic scriptures.

He believes that the Qur'an is sufficient since it contains guidelines for addressing life's realities in its verses. Shahrur created the Theory of Limits to implement his proposal (Nazariyyat al-Hudud). Shahrur attempts to integrate the muhkamat al-Qur'an texts into the reality of life with its constraints through this Theory of Limits. He asserts that the laws of the Qur'an are adaptable and can be employed in any situation, regardless of place or time. As long as it stays within certain bounds and does not go beyond them, the law permits it to be done regardless of the state of the community

The theory of limits consists of a lower limit ( *al-hadd al-adna*/minimum) and an upper limit ( *al-hadd al-a'la*/maximum). There are six forms of application of this theory of limits in the study of legal verses, namely as quoted by M. Amin Abdullah from Shahrur's thoughts (see Table 3): ^25^

**Table 3.** Theory of Limits applications.

No	Theory of Limits	Application
1.	Which only has a lower limit.	This applies to women who may be married (QS. [4]: 22-23), one must not marry former wives of fathers, mothers, daughters, sisters, paternal and aunts, brother's daughters, sister's daughters, foster-mother, foster-sister, mothers in law, stepdaughters under guradianship, wives of sons, or two sisters at the same time Women's clothing (QS. [4]: 31). Applies to cover *juyub*, including cleavage under armpit, body parts, genitals only. There are no other intimate parts (aurat).
2.	Which has both an upper and a lower limit.	Applies to polygamy (QS. [4]: 3). Upper/maximum and lower/minimum limit on one case. For Polygamy, limit for quantity (four wives), and limit for quality (the second, third and fourth wives are widows with children).
3.	The provisions of the lower and upper limits are at one point or there is no other alternative and cannot be less or more.	Applies to the law of adultery with a hundred lashes (QS. [24]: 2). On adultery, the lower and upper limit at one point. Men and women guilty of adultery or fornication lash each one of them with a hundred lashes.
4.	The provisions that have a lower and an upper limit at the same time, but both cannot be exceeded; if exceeded. it means you have violated God's rules.	Applies to male and female relationships. If a man and a woman commit acts of approaching adultery but have not committed adultery, then both of them have not fallen within the limits of Allah's *hudud.*
5.	Which has an upper and lower limit, where the upper limit is positive and cannot be exceeded, while the lower limit is negative and can be exceeded.	Applies to material relationships among humans (both men and women). The upper limit is positive in the form of usury, while the lower limit is negative in the form of *zakat* (alms).

The six forms of the theory of limits proposed by Shahrur above have an impact on the *istinbath* of Islamic law. We take the example of the first theory of limits which only has a lower limit, namely regarding women's clothing and intimate parts. When interpreting QS. [24]: 31, "Or children who are still unaware of women's intimate parts *”.* ^26^ According to Shahrur, intimate parts are what make a person ashamed when seen, and intimate parts are not related to *halal* and *haram*, both from near and far.

He gave an example, “If someone is bald and doesn't like people seeing his bald head, then he will wear wigs, because he considers his bald head to be an intimate part.” Then he quoted the hadith of the Prophet, “Whoever covers the intimate parts of a believer, surely Allah will cover his intimate parts.” He commented, covering the intimate parts of the believer in the hadith, does not mean putting clothes on them so that they are not visible. Then he concluded that *aurat* (intimate parts) stems from shame, namely a person's dislike when something is seen, both from their body and behavior. Being shy according to him is relative, changing according to customs, times, and places. ^27^

Therefore, when there is a verse that tells to wear the hijab in the QS. [33]: 59, “ *O Prophet! Ask your wives, daughters, and believing women to draw their cloaks over their bodies. In this way it is more likely that they will be recognized ˹as virtuous˺ and not be harassed.*” ^28^ He interprets that this verse is in the form of teaching, not sharia, and was revealed in Medina which indicates that it must be understood temporally with the aim of security from two disturbances, namely natural or weather disturbances and social disturbances that adapt to local traditions so as not to invite ridicule. ^29^

So Shahrur's conclusion for the hijab has a maximum and minimum limit. The maximum limit is to cover the entire body except the face and palms, while the minimum limit is that which only covers the *juyūb* which according to him includes the cleavage, body parts under the armpits, genitals, and buttocks. Apart from these, it does not include intimate parts and only adapts to community traditions. ^30^

Next, Shahrur also interpreted the verse, “ *Let them not show their adornments, except what normally appears*” to mean there are two kinds of jewelry for women: object jewelry and place jewelry. ^31^ Object jewelry is, for example, clothing and accessories, while place jewelry is the entire woman's body which is commonly seen as the abdomen, back, head, and legs. So, all parts of the body may be exposed based on the verse above. The hidden ones are the *juyub* (pubic area, buttocks, armpits, and breasts). In other words, a woman who only wears underwear out of the house is not considered to have violated Allah's provisions. Showing a stomach piercing is also permissible.

Moreover, he stated that a woman's vital intimate parts (armpits, breasts, and genitals) may be revealed to the seven groups of men mentioned in the QS. [24]: 31, namely brothers, fathers, daughters of a sibling, sons of a sibling, parents of one’s wives and their children. His opinion states that a Muslim woman may be completely naked in front of these men. He said, “If parents see their daughter completely naked, then it is not said that it is haram, but only a disgrace.” ^32^

He even said that the relationship between teenagers of the opposite sex without marriage, or living together is “ *halal*”. Shahrur said that if it is in accordance with their will, without a contract, or without being accompanied by a *sheikh* or without permission, then it is lawful. He stated, “Read the Book of Allah, don't be afraid of it; all of you can do that without intermediaries and without teachers, and promiscuity is lawful, provided there is agreement between the two parties,” he said. He also stated that promiscuity between a man and a woman is a substitute for marriage, and without a written contract, is “halal, and in accordance with *shar'i.*” ^33^

The second example of the theory of limits, which only has an upper limit, is regarding the verse of cutting off hands for thieves. Shahrur argued that the words *qata'a* could mean physical or non-physical cutting. By looking at the basis of the word qata'a, it turns out to have many meanings and not all meanings refer to physical cutting. In addition, in the Qur’an not all the words *qata'a* mean physical cutting. An example of *qata'a* which means physical cutting is found in QS Al-Maidah: 33, ^34^ while the meaning of not physical cutting is in QS. Ali-Imran: 127, ^35^ QS. Al-anfal: 7, ^36^ and QS. Al-Baqarah: 27. ^37^

It is at this stage that Shahrur then concludes that the words *qata'a* in the context of theft can be interpreted as physical or non-physical cutting. By looking at the *maslahah* (the aims and objectives in Islamic law) between physical and non-physical cutting, Shahrur considers that physical cutting in the verse is the maximum law (upper limit) that can be set, while non-physical cuts, for example, putting the perpetrator in prison. This means that in this verse the concept of *hudud al-a'la* (upper limit) applies and the human *ijtihad* is under the *hudud al-a'la.*

Further, the third example of the theory, which has both an upper and a lower limit, that is, the minimum and maximum limits have been set by the Qur’an, while ijtihad is in between the two minimum and maximum limits. For example: QS. An Nisa verse 11, concerning the Division of Inheritance. The maximum limit for men is twice that of women, while the minimum limit for women is 0.5 of men. *Ijtihad* moves between the two maximum and minimum limits by looking at the various aspects that exist.

Regarding polygamy, Shahrur is very strict. With the Theory of Limits, there are two important limitations of terms, namely the limitation on quantity ( *al-Had al-Kamy*), which is four wives, and the limitation on quality ( *al-Had al-Kalfy*), namely the second, third, and fourth wives are widows who have children. Shahrur allows polygamy under two conditions, namely first, the second, third, and fourth wives are widows with children whose husbands left them. Second, the husband must have an uneasy feeling that he will not be able to deal justly with his children. If the two conditions above are not met, polygamy will fail. This means that the prospective wife must be a widow, not a virgin and must have children, as well as the prospective husband must be fair in all aspects, especially the social aspects of life/society. Shahrur applies these two conditions based on the structure of language norms in the Qur'an Surah an-Nisa verse 4. Shahrur takes these two conditions based on the “structure of language rules” in the word of Allah “And if you are afraid you shall not be able to deal justly with the orphans, then marry two, three or four women whom you like.” Shahrur also sees how Allah honors a widow by using the gentle words “ *ma tabalakum*” (women you like) instead of “ *mashi'tum min an-nisa*” which is respect for marriage.

Regarding the law of adultery, which falls into in the fourth limit theory, the provisions of the lower and upper limits are at one point or there is no other alternative and there can be no more or less. This means that the provision of the maximum limit is also the minimum limit, so that *ijtihad* is not possible to take heavier and lighter laws. For example: QS. An-Nur: 2, regarding the punishment for adultery. In that verse the punishment for adultery is a maximum and a minimum limit at the same time, because in that verse there is an order not to do “ *ra'fah*”, which means there is no waiver. Furthermore, regarding the fifth limit theory, a provision that has a lower and upper limit at the same time, but both cannot be exceeded; if exceeded it means violating God's rules, which applies to male and female relationships. If a man and a woman commit acts approaching adultery but have not committed adultery, then both of them have not fallen within the limits of Allah's hudud.

Finally, the rule which has an upper and lower limit, where the upper limit is positive and cannot be exceeded, while the lower limit is negative and can be exceeded applies to material relationships among human beings, both men and women. The upper limit is positive in the form of usury, while the lower limit is negative in the form of *zakat* (alms). The upper limit that cannot be exceeded is usury, the lower limit that can be exceeded is *zakat* ( *zakat* as a negative limit because it is the minimum limit for assets that must be paid). In this case, *zakat* can be exceeded by charity, while usury cannot be exceeded because it is an upper limit that cannot be exceeded.

This study is limitated as it has only discussed women based on Shahrur's theory of limits as tool of analysis, while the book *Nahw Ushūl al-Jadīdah Li al-Fiqh al-Islāmy; Fiqh al-Mar’ah.* This study limitation also raises opportunities for future researchers to conduct studies on other content such as inheritance (الإرث) and testament (وصية).

## Conclusion

The term millennial commentary of Mohamammad Shahrur, in my humble opinion, is quite appropriate after reading Shahrur's views on commentary, which are relevant to be applied to the millennial spirit of this era, an era where all forms of culture and lifestyle meet and influence each other. In this context, Shahrur himself agreed that Islamic legal products must be able to synergize with the socio-cultural context, including the times. Shahrur finally came to the conclusion that the outcome of Islamic law is greatly dependent on the socio-cultural setting thanks to his study's language approach. In order to bring the texts of the Qur'an into alignment with the realities of society everywhere and at any time, Shahrur analyzes the need for reinterpreting the verses of the Qur'an that form the foundation of fiqh (Islamic Jurisprudence). Shahrur mistrusted the Sunnah al-Nabawiyyah as a source of law due to his emphasis on the Qur'anic scriptures. He believes that the Qur'an is sufficient since it contains guidelines for addressing life's realities in its verses. Shahrur created the Theory of Limits to implement his proposal (Nazariyyat al-Hudud).

Shahrur attempts to integrate the muhkamat al-Qur'an texts into the reality of life with its constraints through this Theory of Limits. He asserts that the laws of the Qur'an are adaptable and can be employed in any situation, regardless of place or time. The community's state falls within these bounds, and the law permits it to be done as long as it doesn't go beyond the predetermined.

## Data Availability

Harvard dataverse: Data research of Saifuddin Herlambang, https://doi.org/10.7910/DVN/BRATPU, Harvard Dataverse, V4. ^38^ This project contains the following underlying data:
‐ notes 1. pdf ‐ notes 2. pdf ‐ notes 3. pdf This project contains the following extended data:
‐ Komisi etik.pdf (Letter of research ethical commission from The Institute for Research and Community Service (Lembaga Penelitian dan Pengabdian Kepada Masyarakat/LP2M), Pontianak State Institute for Islamic Studies (in Indonesian)) Data are available under the terms of the Creative Commons Zero “No rights reserved” data waiver (CC0 1.0 Public domain dedication).

## Footnotes

^1^
Uta, ‘Masih ada Diskriminasi Gender [Gender Discrimination Still Exists].’, Media Indonesia (Tuesday, Oct 2018), http://mediaindonesia.com/read/detail/189401-masih-ada-diskriminasi-gender.

^2^
Yuli Saputra, ‘Women's Ulema Congress: Straightening the Discrimination Against Women’, *Rappler.Com* (27 Apr 2017), https://www.rappler.com/indonesia/berita/168095-meluruskan-paham-diskriminatif-perempuan.

^3^
Husein. Indonesian Women's Ulema Congress at the Kebon Jambu Al-Islamiy Babakan Islamic Boarding School, Ciwaringin Cirebon, Interview on Wednesday 26 April 2017.

^4^
Ministry of Religious Affairs of the Republic of Indonesia, *Al-Qur’an dan Terjemahan* (Madinah: Qadim Al-Haramain Al-Syarifain, 1980).

^5^
Husein. Indonesian Women's Ulema Congress at the Kebon Jambu Al-Islamiy Babakan Islamic Boarding School, Ciwaringin Cirebon, Interview on Wednesday 26 April 2017.

^6^
Women's Ulema Congress: Straightening the Discrimination Against Women’.

^7^
Richard C. Martin (ed.) Zakiyuddin Baidhawy (tran.), ‘Islam and Religious Studies: An Introduction’, in Approaches to Islam in Religious Studies (Yogyakarta: Pustaka Pelajar, 2001), p. 1.

^8^
Jose Casanova, *Public Religion in the Modern World* (Chicago and London: The University of Chicago Press, 1994).

^9^
Muḥammad ʻ Ā bid al-Jabiri, *Takwīn al-ʻaql al-ʻArabī*, vol. 1 (Bayrūt: Markaz Dirāsāt al-Waḥdah al-‘Arabīyah, 2014); Muḥammad ʻ Ā bid al-Jabiri, *Bunyah al-Aql al-‘Arabi: Dirasah Tahliliyah Naqdiyah li Nudzum al-Ma’rifah fi al-Tsaqafah al-‘Arabiyah* (Bayrūt: al-Markaz al-Tsaqafi al-Arabi, 1991).

^10^
Khaled M. Abou El-Fadl, *Atas Nama Tuhan; Dari Fiqh Otoriter ke Fiqh Otoritatif* (Jakarta: Serambi Ilmu Semesta, 2004).

^11^
Abdul Karim Soroush, *Menggugat Otoritas dan Tradisi Agama [Challenging Religious Authority and Traditions]* (Bandung: Mizan, 2002).

^12^
Abdullah Saeed, *Interpreting the Qur’an: Towards a Contemporary Approach* (Oxon: Routledge, 2005); Abdullah Saeed, *The Qur’an: An Introduction* (London and New York: Routledge, 2008).

^13^
Muhammad Shahrur, *Nahwa Usul Jadidah lil Fiqh Islamy: Fiqh al-Mar’ah* (Damascus: Al-Ahali li-t-Tibaah, 2000); Muhammad Shahrur, *Al-Kitab wa al-Qur’an: Qira’ah Muasirah* (Damascus: Al-Ahaliy li at-Tabaah wa an-Nasyr wa at-Tawzi’, 1990).

^14^
Richard C. Martin (ed.) Zakiyuddin Baidhawy (tran.), ‘Islam dan Studi Agama: Sebuah Pengantar’.

^15^
A Lutfi Syaukani, ‘Tipologi dan Wacana Pemikiran Arab Kontemporer’ [Typology and Discourse of Contemporary Arabic Thought], *Jurnal Paramadina*, no. 2 (1998).

^16^
E. Paler, Richard. *Hermeneutics: Theory of Interpretation in the Thoughts of Schleiermacher, Dilthey, Heidegger, and Gadamer. Translated by. Stephanus Aswar Herminarko. IRCiSoD, Yogyakarta: First Edition, July, 2022.*p.92.

^17^
E. Paler, Richard. *Hermeneutics: Theory of Interpretation in the Thoughts of Schleiermacher, Dilthey, Heidegger, and Gadamer. Translated by. Stephanus Aswar Herminarko. IRCiSoD, Yogyakarta: First Edition, July, 2022.*p.92.

^18^
Byrne M. Hermeneutics as a methodology for textual analysis. AORN J. 2001 May;73(5):968-70. doi: 10.1016/s0001-2092 (06)61749-3. PMID: 11378953.

^19^
Austgard, K. (2012). Doing it the Gadamerian way–using philosophical hermeneutics as a methodological approach in nursing science. *Scandinavian Journal of Caring Sciences*, *26*(4), 829-834.

^20^

*Ibid.*

^21^
For a more complete explanation of Muhammad Shahrûr's biography, please refer directly to Shahrur's official website, namely: http://www.shahrour.org.

^22^
Peter Clark, ‘The Shahrorur’s Phenomenon; A Liberal Islamic Voice From Syiria’, *Islam and Christian-Muslim Relation*, vol. VII, no. 3 (1996), p. 339.

^23^
Syahrur, M. (2004). Principles and Basis of Contemporary Al-Qur'an Hermeneutics. Sahiron Syamsuddin (translation) *Yogyakarta: elSaq.* Cet. Ke-I, p. 65.

^24^
Khudori Soleh dkk, ‘Mohammad Shahrur: Teori Batas’ [the Theory of Limits], in *Pemikiran Islam Kontemporer* (Yogyakarta: Jendela, 2003), p. 296.

^25^
M. Amin Abdullah, *Neo Ushul Fiqih Menuju Ijtihad Kontekstual* (Yogyakarta: Fakultas Syari’ah Press dan Forum Studi Hukum Islam [Faculty of Shari'a Press and Islamic Law Study Forum], 2004), pp. 156–8.

^26^
Ministry of Religious Affairs of the Republic of Indonesia, *Al-Qur’an dan Terjemahan.*

^27^
Muhammad Shahrur, *Nahwa Usul Jadidah lil Fiqh Islami: Fiqh al-Mar’ah*, p. 370.

^28^
Ministry of Religious Affairs of the Republic of Indonesia, *Al-Qur’an dan Terjemahan.*

^29^
Muhammad Shahrur, *Nahwa Usul Jadidah lil Fiqh Islami: Fiqh al-Mar’ah*, pp. 372–3.

^30^

*Ibid.*, pp. 376–8; Muhammad Shahrur, *Al-Kitab wa al-Qur’an: Qira’ah Muasirah*, p. 607.

^31^
Muhammad Shahrur, *Al-Kitab wa al-Qur’an: Qira’ah Muasirah*, p. 606.

^32^

*Ibid.*, p. 607.

^33^
Harda Armayanti, ‘Mengenal Shahrur dan Teori Batasnya’, *Kajian Timur Tengah [*Mengenal Shahrur dan Teori Batasnya’, *Kajian Timur Tengah]* (Desember 2010), https://kajiantimurtengah.wordpress.com/2010/12/04/mengenal-Shahrur-dan-teori-batasnya/., yang ia kutip dari *http://www.Hidayatullah.com* publikasi Selasa, 29 Januari 2008.

^34^
Verily, the recompense for those who fight against Allah and His Messenger and cause mischief in the earth is only that they are killed or crucified, or their hands and feet are cut off in exchange for reciprocity, or are banished from the land (where they live). That is (as) an insult to them in this world, and in the Hereafter they will have a great torment. Kementerian Agama Republik Indonesia [Ministry of Religion of the Republic of Indonesia], Al-Qur'an and its Translation, *Al-Qur’an dan Terjemahan.*

^35^
(Allah helps you in the battle of Badr and gives reinforcements) to destroy a group of disbelievers, or humble the rest, causing them to withdraw in disapointment. *Ibid.*

^36^
And (remember), when Allah promised you that one of the two groups (which you faced) was for you, while you wanted that which did not have the strength of a weapon was for you, and Allah wanted to justify the truth with His verses and destroy Unbelievers. *Ibid.*

^37^
Who break the covenant of Allah after contracting it and sever that which Allah has ordered to be joined and cause corruption on earth. It is those who are the losers. *Ibid.*
