# Supplementary material for: Interpretasi milenial Muhammad Shahrur tentang isu-isu perempuan
Source: F1000Res. 2024 Sep 26;12:39. Originally published 2023 Jan 10. [Version 4] doi: 10.12688/f1000research.125653.4 (PMC11269973; doi:10.12688/f1000research.125653.4)
Supplement: Peer review history

# Reviewer report: Reviewer response for version 3

**DOI:** 10.5256/f1000research.163004.r293056

**Published:** 2024-07-24

**Authors:** Muhammad Sofwan

**Affiliations:** 1 Univeristas Jambi, Jambi, Indonesia

Some improvements can be made

 a. The methodology part needs to be more defined and organized. Give a clear explanation of each stage in the research process.

 b. Steer clear of unduly broad claims. Don't just say, "This was an exploratory bibliographic study," for example; instead, detail the precise techniques employed for gathering and analyzing the data.

 c. Outline the two books' selection criteria as well as the particular chapters that make up each one. Justify the selection of these specific chapters.

 d. Provide a more convincing defense of the application of descriptive-hermeneutic analysis. Describe the reasons this approach is appropriate for your research and how it advances your goals.

 a. Include a succinct literature review in the methodology section to place your study in the perspective of earlier studies. Emphasize how your method differs from previous research on related subjects.

 f. Describe the steps involved in gathering the data, such as how the texts were logged, bookmarked, and examined.

 g. Go into further detail about the hermeneutic analysis procedure. Explain your interpretation of the texts and the particular methods you employed to guarantee a thorough examination.

 g. Discuss Shahrur's theory of limits in further detail. Give more thorough justifications and instances to support his points of view.

 i. To provide a more thorough examination, compare and contrast Shahrur's interpretations with those of other Islamic thinkers. This will assist in highlighting Shahrur's approach's significance and originality.

 j. Place Shahrur's interpretations in the context of modern discussions on women's issues and larger Islamic thought. Talk about how his opinions coincide with or deviate from popular Islamic interpretations.

 k. Assess Shahrur's interpretations critically. Talk about the possible advantages and disadvantages of his methodology as well as the ramifications for modern Islamic thought. l. Respond to any possible objections to Shahrur's viewpoints and offer a fair discussion that takes into account both the arguments in favor of and against them.

 m. Provide a succinct and clear summary of your study's key findings. Emphasize the most important conclusions drawn from examining Shahrur's perspectives on women's challenges.

 n. Talk about the findings' wider ramifications for Islamic philosophy and the current debates over women's rights. Describe how Shahrur's interpretations might affect research and practice in the future.

 o. Recognize the constraints on your research. Talk about how these restrictions might have impacted your results and make recommendations for further study.

 p. Outline particular inquiries or study areas that could expand on your conclusions and go deeper into the ramifications of Shahrur's notion of limitations.

Is the work clearly and accurately presented and does it cite the current literature?

Yes

If applicable, is the statistical analysis and its interpretation appropriate?

Not applicable

Are all the source data underlying the results available to ensure full reproducibility?

Partly

Is the study design appropriate and is the work technically sound?

Yes

Are the conclusions drawn adequately supported by the results?

Partly

Are sufficient details of methods and analysis provided to allow replication by others?

No

Reviewer Expertise:

Education and statistics

I confirm that I have read this submission and believe that I have an appropriate level of expertise to confirm that it is of an acceptable scientific standard, however I have significant reservations, as outlined above.

# Reviewer report: Reviewer response for version 2

**DOI:** 10.5256/f1000research.153259.r209742

**Published:** 2023-11-22

**Authors:** Ali Akbar

**Affiliations:** 1 The University of Melbourne, Melbourne, Victoria, Australia

The methodology does not seem to be carried out in an academic format. You should not say which library was used and which keywords were searched for the purpose of writing the article. I suggest the author to change the method. In terms of data collection, you do not need to say each book consisted of how many chapters. These should all be fixed. Another issue with the article is that it requires a comprehensive literature review. There are some studies and articles written about Shahrur (in English). The author should refer to these works (especially about women’s rights) and then articulate how his/her article contribute to the literature of the field.

 The section on analysis starts with hermeneutics. The author should acknowledge how, and to what degree, Muslim scholars during the modern period have engaged with hermeneutics (for this the author should refer to: Ali Akbar, “Philosophical Hermeneutics and Contemporary Muslim Scholars’ Approaches to Interpreting Scripture”, *Philosophy & Social Criticism* 47 (5), 2021: 587-614). Overall, hermeneutics should be explained in greater details.

 In p.7, the author claims, “Islamic law is greatly influenced by the socio-cultural setting”. This is a methodology applied by many contemporary Muslim scholars with a reformist mindset. The author should explain this methodology. Shahrur’s approach must be explained within the contextualist framework, so that his contribution can be understood better. I suggest the author to refer to Ali Akbar’s works, in particular: “Towards a humanistic approach to the Quran: new direction in contemporary Islamic thought”, *Culture and Religion *20(1): 82-103; “Fazlur Rahman’s Influence on Contemporary Islamic Thought”, *The Muslim World* 110(2):129-153; *Contemporary Perspectives on Revelation and Quranic Hermeneutics: An Analysis of Four Discourses*, Edinburgh, Edinburgh University Press, 2020. Of course, detailed analysis of the ideas of other liberal scholars are not required, but the author is expected to refer to these works in order to show readers that similar approaches have been presented by other reformist Muslim scholars (This is what the author did in p.8 where he/she referred to other scholars such as Qaradawi and Hamka when explaining Shahrur’s idea on hijab).

 Overall, while the section on Shahrur reads fine, the author is expected to refer to some sources (such as above) to make his/her argument and Shahrur’s contribution clearer.

 Finally, there are some sentences throughout the article which do not read well and I recommend the authors to restructure them. Overall, the article requires a professional proofread.

Is the work clearly and accurately presented and does it cite the current literature?

Partly

If applicable, is the statistical analysis and its interpretation appropriate?

Not applicable

Are all the source data underlying the results available to ensure full reproducibility?

Partly

Is the study design appropriate and is the work technically sound?

Yes

Are the conclusions drawn adequately supported by the results?

Yes

Are sufficient details of methods and analysis provided to allow replication by others?

Partly

Reviewer Expertise:

Islamic studies

I confirm that I have read this submission and believe that I have an appropriate level of expertise to confirm that it is of an acceptable scientific standard, however I have significant reservations, as outlined above.

## References

[1] : Philosophical hermeneutics and contemporary Muslim scholars’ approaches to interpreting scripture. *Philosophy & Social Criticism* .2021;47(5) : 10.1177/0191453720931912 587-614 10.1177/0191453720931912

[2] : Towards a humanistic approach to the Quran: new direction in contemporary Islamic thought. *Culture and Religion* .2019;20(1) : 10.1080/14755610.2018.1532919 82-103 10.1080/14755610.2018.1532919

[3] : Fazlur Rahman’s Influence on Contemporary Islamic Thought. *The Muslim World* .2020;110(2) : 10.1111/muwo.12334 129-153 10.1111/muwo.12334

[4] : Contemporary Perspectives on Revelation and Quranic Hermeneutics: An Analysis of Four Discourses. *Edinburgh, Edinburgh University Press* .2020;

# Reviewer report: Reviewer response for version 2

**DOI:** 10.5256/f1000research.153259.r194892

**Published:** 2023-10-03

**Authors:** Nur Zainatul Nadra Zainol

**Affiliations:** 1 Department of Islamic Studies, Universiti Tun Hussein Onn Malaysia, Batu Pahat, Johor, Malaysia

The Author have done the correction based on the comments.

Is the work clearly and accurately presented and does it cite the current literature?

Yes

If applicable, is the statistical analysis and its interpretation appropriate?

Partly

Are all the source data underlying the results available to ensure full reproducibility?

Partly

Is the study design appropriate and is the work technically sound?

Yes

Are the conclusions drawn adequately supported by the results?

Partly

Are sufficient details of methods and analysis provided to allow replication by others?

Yes

Reviewer Expertise:

Islamic Studies; Quranic Interpretation; Modern Muslim Thinker

I confirm that I have read this submission and believe that I have an appropriate level of expertise to confirm that it is of an acceptable scientific standard.

# Reviewer report: Reviewer response for version 1

**DOI:** 10.5256/f1000research.137984.r177220

**Published:** 2023-07-11

**Authors:** Nur Zainatul Nadra Zainol

**Affiliations:** 1 Department of Islamic Studies, Universiti Tun Hussein Onn Malaysia, Batu Pahat, Johor, Malaysia

This article is well-constructed and carefully written to understand Muhammad Shahrur's interpretation on women issues. However, it requires some amendments for the paper to be accepted. The abstract should include a statement about interpretation on ayah muhkamat must along with fiqh mazahib.

 Data interpretation on the 1st women issues; relationships between teenagers of the opposite sex without marriage or living together is "halal" if it follows their will, without a contract, being accompanied by syecih or permission - this is controversial and must do comparison with other opinions. 

 The references only have 2 in recent 5 years, requiring more to fit into the state-of-the-art literature. This paper is considered to be indexed after amendments are made.

Is the work clearly and accurately presented and does it cite the current literature?

Yes

If applicable, is the statistical analysis and its interpretation appropriate?

Partly

Are all the source data underlying the results available to ensure full reproducibility?

Partly

Is the study design appropriate and is the work technically sound?

Yes

Are the conclusions drawn adequately supported by the results?

Partly

Are sufficient details of methods and analysis provided to allow replication by others?

Yes

Reviewer Expertise:

Islamic Studies; Quranic Interpretation

I confirm that I have read this submission and believe that I have an appropriate level of expertise to confirm that it is of an acceptable scientific standard, however I have significant reservations, as outlined above.

## References

[1] : Structure and Qur’anic Interpretation: A Study of Symmetry and Coherence in Islam's Holy Text. *Hitit Üniversitesi Sosyal Bilimler Enstitüsü Dergisi* .2018; 10.17218/hititsosbil.457258 10.17218/hititsosbil.457258

# Response

**Published:** 2023-07-18

**Authors:** Saifuddin Herlambang

**Affiliations:** State Institute for Islamic Studies (IAIN) Pontianak, Indonesia

Dear reviewer, thank you for your suggestion. This additional text written on the text on appropriate section.

 This article presents a study of interpretation in tafsir. Interpreters have been grouped into two categories: modern/progressive interpreters and conventional interpreters. Syahrur and Amena Wadud are prominent examples of progressive modern interpreters. It is important to clarify that this study remains independent of any specific madhhab, focusing solely on tafsir analysis 

 All interpreters and scholars except Syahrur have agreed that *musakanah (samen leven)* are “haram”. Muhammad Kholid holds the view that Syahrur's methodology and epistemology are full of inconsistency and problems. This interpretation departs from the mainstream consensus of the majority of Muslim scholars. (abstract)

 In response to Abdul Aziz's dissertation on Syahrur's stance regarding the permissibility of sex outside of marriage, Deputy Chairman of the Indonesian Ulema Council (MUI), Yunahar Ilyas, asserted that such a view contradicts the teachings of the Qur'an. Syahrur's perspective suggests that the roles performed by slaves in ancient times served as a substitute for certain functions, including sexual relations, household assistance, and work relations. He draws support for the function of sexual relations from Surah Al-Mukminun verses 5 and 6, the function of household assistance from Surah An-Nuur verse 58, and the function of work relations from Surah An-Nahl verse 71.

 Hamka and Yusuf Qardhawi hold the belief that a woman's private parts encompass her entire body, with the exception of the face and palms. In contrast, Syahrur presents a distinction, dividing it into two categories: the lower limit comprises the concealed genitalia, causing embarrassment if exposed, while the upper limit includes the entirety of a woman's body, excluding the face and palms. On the subject of hijab, Quraish Shihab thinks that it is not obligatory but rather a recommended practice.

 Additional for State of the art: 

 Rosyada's research focused on the subject of polygamy and justice according to Syahrur (2019). Meanwhile, Hidayat conducted a comparative analysis, examining Wahbah Az-Zuhaili's perspective alongside Syahrur's views on polygamy (2020). In addition, Hadi explored the concept of *milkul yamin* from the perspective of ushul fiqh, focusing on Syahrur's insights (2019). Building on this theme, Husna further investigated the boundaries of women's genitals, drawing from Syahrur's book (2021). Auliya and Gazali contributed to the discussion by providing a comprehensive review of the concept of female genitalia based on Syahrur's boundary theory (2020). Similarly, in 2021, Fathony and Hamid delve into the reconstruction of Syahrur's thoughts on women's private parts in the Indonesian Archipelago. Lastly, in 2022, Ramadana undertook a comparative study, analyzing the perspectives of Fatima Mernissi, Quraish Shihab, and Syahrur, offering insights into their perpectives.
